# Measuring the Speed of Aging across Population Subgroups

**DOI:** 10.1371/journal.pone.0096289

**Published:** 2014-05-07

**Authors:** Warren C. Sanderson, Sergei Scherbov

**Affiliations:** 1 Department of Economics, Stony Brook University, Stony Brook, New York, United States of America; 2 World Population Program, International Institute for Applied Systems Analysis, Laxenburg, Austria; 3 Wittgenstein Centre for Demography and Global Human Capital (International Institute for Applied Systems Analysis, IIASA; Vienna Institute of Demography of the Austrian Academy of Sciences, VID/ÖAW; Vienna University of Economics and Business, WU), Laxenburg/Vienna, Austria; Hunter College, City University of New York (CUNY), CUNY School of Public Health, United States of America

## Abstract

People in different subgroups age at different rates. Surveys containing biomarkers can be used to assess these subgroup differences. We illustrate this using hand-grip strength to produce an easily interpretable, physical-based measure that allows us to compare characteristic-based ages across educational subgroups in the United States. Hand-grip strength has been shown to be a good predictor of future mortality and morbidity, and therefore a useful indicator of population aging. Data from the Health and Retirement Survey (HRS) were used. Two education subgroups were distinguished, those with less than a high school diploma and those with more education. Regressions on hand-grip strength were run for each sex and race using age and education, their interactions and other covariates as independent variables. Ages of identical mean hand-grip strength across education groups were compared for people in the age range 60 to 80. The hand-grip strength of 65 year old white males with less education was the equivalent to that of 69.6 (68.2, 70.9) year old white men with more education, indicating that the more educated men had aged more slowly. This is a constant characteristic age, as defined in the Sanderson and Scherbov article “The characteristics approach to the measurement of population aging” published 2013 in *Population and Development Review*. Sixty-five year old white females with less education had the same average hand-grip strength as 69.4 (68.2, 70.7) year old white women with more education. African-American women at ages 60 and 65 with more education also aged more slowly than their less educated counterparts. African American men with more education aged at about the same rate as those with less education. This paper expands the toolkit of those interested in population aging by showing how survey data can be used to measure the differential extent of aging across subpopulations.

## Background

Subgroups of populations can age at different rates. An assessment of these subgroup differences could potentially aid in improving the health of people in groups that age more quickly. Differences in the extent of aging across subgroups may also help in explaining subgroup differences in forward-looking behaviors.

The study of aging differences across subgroups necessarily involves the use of characteristics of people other than their chronological age. In estimating how much faster people in one subgroup have aged relative to another, we must specify the characteristic or characteristics that we use in this assessing this. In this article, we provide a procedure for studying differences in subgroup aging using characteristics that can be found in datasets such as CHARLS [1], ELSA [2], HRS [3], KLoSA [4], SAGE [5], and SHARE [6], which provide a rich array of variables that can be used to define subpopulations. We implement the procedure, in this paper, using data on a particular characteristic, hand-grip strength, but it could be implemented using many different characteristics as well.

Hand-grip strength is a measure of upper body strength that has been widely studied and that has been consistently shown to be a good predictor of future morbidity and mortality. This makes it a particularly useful characteristic in the study of differences in subgroup aging.

Bohannon [7] reviewed 45 articles on the relationship between hand-grip strength and later mortality and morbidity outcomes. The articles included data from both healthy individuals and those with serious health conditions. Most of the studied subjects were middle-aged or older. Mortality outcomes were the subject of 24 papers. Disability was the subject of 9, and complications of surgeries or length of hospital stays was investigated in 12. A variety of other health related outcomes were also studied. Sixteen of the studies covered community-dwelling individuals, most of whom were healthy. In all the studies, low hand-grip strength was associated with greater levels of mortality, morbidity, worse health outcomes, or longer hospital stays.

Cooper et al. [8] reviewed studies of community dwelling people that investigated the relationship between measures of physical capacity such as hand-grip strength, walking speed, standing balance, and chair rise speed with subsequent mortality. Research on hand-grip strength and mortality included studies with follow-up periods from less than 5 years to over 20. Twenty-three studies on hand-grip were analyzed using random-effects meta-analysis models and a statistically significant negative relationship between hand-grip strength and subsequent mortality was found.

Cooper et al. [9] reviewed similar studies with subsequent fractures, cognitive decline, cardiovascular disease, and hospitalization or institutionalization as outcomes. In 9 studies of the relationship between hand-grip strength and subsequent fractures, 4 presented strong evidence that weaker hand-grip strength was associated with a greater risk of fracture, 3 presented weak evidence, and 2 showed no relationship. In 3 studies of the association between hand-grip strength and subsequently cognitive decline, all published strong evidence for weaker hand-grip strength being associated with future cognitive decline. In 3 studies related to cardiovascular disease, 2 studies provided strong evidence of an association, while 1 presented only weak evidence. There was 1 study of hand-grip strength and future hospitalization or institutionalization. The evidence of an association there was weak.

Ling et al. [10] reports on a study of 555 people in Leiden, Netherlands who were 85 years old when enrolled between September, 1997 and September, 1999. Survivors were followed through February, 2008 using register data. Control variables included important comorbidities, functional status as measured by ADLs and IADLs, depression, and mental state. The main result was that study participations with relatively low hand-grip strength at age 85 had a statistically significantly higher all-cause mortality rate.

In Chen et al. [11], 558 men 75+ years old in a nursing home in Taiwan were followed for 3 years. Low hand-grip strength was statistically significantly associated with the risk of infection-related death.

Rantanen et al. [12] reported on a study of 2,239 men who were born before June, 1909 and had hand-grip strength measurements taken between 1965 and 1968 when they were between 56 and 68 years old. The observation period ended in June, 2009 when all the men had either died or reached the age of 100+ years old. When centenarians were compared with those who died at age 79 or earlier, it was found that they were 2.5 times (95% confidence interval (1.23–5.10)) more likely to have had hand-grip strength in the top third on the baseline hand-grip strength distribution. This study also had an observation not found in any of the previous research. Multivariate analysis suggested that mother’s longevity and her offspring’s hand-grip measured at mid-life affected the offspring’s longevity through similar pathways. The causes of this association are unclear. It could be that there is a genetic component to hand-grip strength. Another possibility is that mothers with higher education had higher life expectancies themselves and raised healthier children who subsequently had stronger hand-grips. Evidence consistent with the hypothesis of a connection between parental longevity and hand-grip strength can also be found in Frederiksen et al. [13].

Ortega et al. [14] analyzed data from 1,142,599 Swedish adolescent males born between 1951 and 1976, based on the Swedish Military Conscription Register. At that time all Swedish young men had to take pre-conscription examinations even if they did not subsequently serve in the armed forces. Only young men with severe handicaps or chronic diseases were exempt from those tests. The young men were followed until the earliest of three outcomes, death, emigration or the end of data collection on December 31, 2006. The median follow-up period was 24.2 years, with a range of 1.0 to 37.3 years. Data were unused if the follow-up period was less than a year (to eliminate those who were very sick when the exams were given), or if the subjects had extreme readings on their height, weight, body mass index, or on their blood pressure. Four outcomes were delineated, all-cause mortality, mortality from cardiovascular disease, cancer and suicide. Using Cox proportional hazard models, the authors showed that lower hand-grip strength, particularly for those with hand-grip strengths below the median, were significantly associated with higher all-cause mortality, higher mortality from cardiovascular disease, and a higher risk of suicide. Hand-grip strength was not associated with the risk of dying of cancer. In an exploratory supplementary analysis, the authors found that low hand-grip strength among the adolescents was also predictive of the development of subsequent psychological problems.

As the studies above show, low hand-grip strength has been shown definitively to predict poor outcomes in a wide variety of mortality, morbidity, and other health outcomes such as lengths of stay in hospitals or rehabilitation centers. The associations have been demonstrated for both younger and older people, for community-dwelling populations and those in institutions, and for people in many different countries. Because of these associations with a wide variety of characteristics that are associated with aging, hand-grip strength is a useful metric for assessing how fast sub-groups of a population have aged.

## Methods

The data that we use here are from the 2006, 2008, 2010, and 2012 waves of the Health and Retirement Survey (HRS) [15]. The HRS is sponsored by the National Institute on Aging (grant number NIA U01AG009740) and is conducted by the University of Michigan. Hand-grip strength was initially collected in a small sample of the HRS participants in 2004, but the later four waves are more comparable in survey design and implementation. Table S1 shows mean hand-grip strength by 5 year age groups from 60–64 to 80–84 cross-classifed by race, gender, and education. Hand-grip strengths were measured in kilograms using a Smedley spring-type dynamometer and are derived as the average of four observations, two for each hand. We investigate the effect of education on hand-grip strength with random-effects panel regressions using the specification:
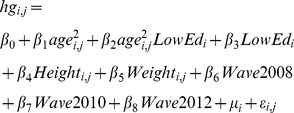
or more compactly 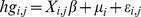.

The dependent variable is the hand-grip strength of person *i* in wave *j*. The independent variables are *age^2^*, a dummy variable indicating that formal education was completed prior to obtaining a high school degree (*LowEd*), the interaction of *age* ^2^ and *LowEd*, height, weight and dummy variables indicating the HRS wave. The person-specific component, 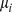, is assumed to be a normally distributed IID random variable which is uncorrelated with the independent variables and with the idiosyncratic random term, 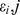. All regressions are run separately for white men, white women, African-American men, and African-American women on data from which extreme outliers were removed. Details about this eliminated cases can be found in Text S1. Information about the independent variables that we used can be found in Table S2. In each of these four groups there are two disjoint panels, one for the years 2006 and 2010 and another for the years 2008 and 2012. We tested specifications linear in age and found that the inclusion of age linearly did not increase the adjusted R^2^ and made the other coefficient estimates less precise. The results of the regressions can be found in Text S2.

Our prefered specification has no correction for selectivity and does not use weights. We did tests for selectivity using the Heckman 2-step procedure and found none (see Text S3). Weights appropriate for hand-grips strength measures are currently available only for 2006 and 2008. Therefore it was impossible to use weights in the panel estimates. We did tests for the sensitivity of our results to the use of weights using the 2006 and 2008 data separately and found that our results regarding age differentials were robust to whether or not weights were used (see Tables S3 and S4).

The predicted mean hand-grip stregth conditional on a set of covariates X^*^ is.
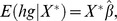
where 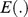 is the expectation operator and 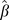 is a vector of estimated regression coefficients. Our interest is in the chronological ages of subpopulations with equivalent mean hand-grip strengths. Holding height, weight, and wave constant and denoting the chronological age of people with low education *age_L_*, for a given set of estimated coefficients, 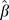, the age of more educated people with the same mean hand-grip strength can be computed as
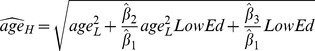



The estimated parameter vector 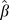 is itself random and therefore 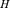 is random, conditional on values of 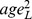 and *LowEd*. Given the variance-covariance matrix of 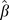, we simulated 1,000 values of  (i = 1,2,3) and the corresponding distributions of 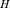. These 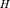 are constant characteristic ages, as defined in Sanderson and Scherbov [16].

## Findings

The means and the 95% confidence intervals for 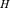 are shown in Table 1 for the four race-gender groups.

**Table 1:** People with the same hand-grip strength based age, by age, gender, race, and education, means and 95% confidence intervals.

	Whites – More Educated		African Americans –More Educated	
Reference Age of Less Educated	Male	Female	Male	Female
60	65.8 (63.9,67.7)	65.7 (63.9,67.3)	57.6 (53.4,61.4)	64.7 (60.5,68.2)
65	69.6 (68.2,70.9)	69.4 (68.2,70.7)	63.4 (60.3,66.3)	68.5 (65.3,71.3)
70	73.4 (72.3,74.5)	73.3 (72.3,74.3)	69.2 (66.5,71.6)	72.3 (69.5,74,8)
75	77.3 (76.4,78,3)	77.2 (76.4,78.1)	74.7 (71.9,77.6)	76.1 (73.3,79.0)
80	81.3 (80.2,82.3)	81.2 (80.2,82.2)	80.3 (76.9,83.9)	80.0 (76.5,83.7)

We can see from Table 1 that white males and females and African-American females with a high school education or more age less rapidly than those with less than a high school education. The advantage associated with more education diminishes as people get older and essentially disappears by age 80. African-American males differ in that no statistically significant differences by education in hand-grip based ages can be found.

## Discussion

Table 1 shows converge in two dimensions. First, educational differentials in hand-grip based ages tend to disappear as people get older. Second, racial differences also disappear as populations age. Selectivity by fraility and perhaps other characteristics likely accounts for a portion of this convergence. There could also be cohort differences in the effects of education. Eighty year olds were educated at an earlier time than 60 year olds and the contents of their educational experiences with respect to health could have been different. The lack of an effect of education for African-American men, while there is one for African-American women in their 60’s needs further study. Roughly speaking, the African-American men and women grew up in similar early health environments and had comparatively similar early educational experiences. Possibly, African-American women with a high school education or more worked in occupations with greater access to health-relevant information or better health insurance compared to African-American men with a similar level of education.

A number of papers have produced biomarker-based biological ages. These have been oriented to studying the ageing process in individuals and not to measuring subgroup differences in health. There is no consensus on the best approach to determining biological age. Levine [17] evaluates a number of these and found that a variant of the Klemera and Doubal [18] method predicts future mortality best. However, the Klemera and Doubal procedure is complex and requires *ad hoc* assumptions. In contrast, the approach put forward here is simple and transparent.

In this paper, we have presented a simple procedure for using a biomarker, hand-grip strength, to produce a comparative measure of aging across population subgroups. Because our measure is a characteristic-based age [16] it is easy to understand, interpret, and communicate to policy-makers.

## Supporting Information

Table S1 **Mean hand-grip strength by 5 year age groups from 60–64 to 80–84 by race, gender, and education, for wave 2006, 2008, 2010, 2012.**
(XLSX)

Table S2 **Data on the independent variables used in the panel regressions.**
(XLSX)

Table S3 **Hand-grip strength based ages with and without weights by 5 year age groups by race, gender, and education for 2006.**
(XLSX)

Table S4 **Hand-grip strength based ages with and without weights by 5 year age groups by race, gender, and education for 2008.**
(XLSX)

Text S1 **Cases included in the estimation.**
(DOCX)

Text S2 **Random effect regression results.**
(DOCX)

Text S3 **Tests for Selection.**
(DOCX)
