# Supplementary material for: Measuring the Speed of Aging across Population Subgroups
Source: PLoS One. 2014 May 7;9(5):e96289. doi: 10.1371/journal.pone.0096289 (PMC4012980; doi:10.1371/journal.pone.0096289)
Supplement: Text S1 — Cases included in the estimation. (DOCX) [file pone.0096289.s005.docx]

**Text S1 Cases included in the estimation.**

We included only:

1. African-Americans and whites

2. People 55 to 90 years old

3. Waves 2006, 2008, 2010, and 2012

4. People with a known level of education

5. People with HRS variable hg_sample = 3 and HRS variable try = 1.

The variable hg_sample divides the sample in each wave into three interview type groups *ex ante*. Three types of interview types are: phone interview, normal face to face interview, and enhanced face to face interview. Hand-grip strength was measured only in enhanced face to face interviews. In wave 2006, half of sample was chosen for enhanced face to face interviews. These people were chosen for face to face interviews in 2010. The other half of the sample was chosen for enhanced face to face interviews in 2008 and they were reinterviewed in 2012. This results in data for two independent panels each generally with two observations. Table A shows the distribution of the hg_sample variable for wave 2010.

Table A. Numbers of people by hg_sample code in 2010 wave (no restrictions)

| **Ex Ante Interview Type** | **Code** | **Number of People** |
| --- | --- | --- |
| Phone | 1 | 5720 |
| Normal Face to Face | 2 | 2609 |
| Enhanced Face to Face | 3 | 8780 |
| Missing | Blank | 5 |

Among the 8,780 people chosen for enhanced face to face interviews in 2010, 2,024 did not participate. Of those, 217 had the enhanced face to face interview, but did not take the hand-grip strength test for a number of reasons, mainly because they had some physical difficulties with taking the test. 1,807 people who were chosen to have the enhanced face to face interview did not have it. Among the original 8,780 people in the sample 23 percent of those living alone did not have the enhanced face to face interview compared with 19 percent of those who were living with someone.

After implementing the five inclusion criteria above, we had 23,917 observations in all four waves combined. We did a first round cleaning by eliminating observations with hand-grip strengths 5 kgs or less (14 people). Among those observations with hand-grip strength over 5 kgs, we eliminated 33 with known heights and weights because their weight was 70 pounds or less or greater than 350 pounds. We also eliminated people who were shorter than 50 inches tall. We also eliminated 1,302 observations where either height, weight or both were unknown. The result is a remaining sample of 22,573 observations.

We dealt with those 22,573 observations in two steps. First we estimated the parameters of our model using random effects panel regression. Q-Q plots suggested that the residuals were not normally distributed at the low end of the distribution. So we deleted around 1 percent of the observations with the lowest residuals and reestimated the model.

The panel regressions were run using the package plm in R.
