# Supplementary material for: Measuring the Speed of Aging across Population Subgroups
Source: PLoS One. 2014 May 7;9(5):e96289. doi: 10.1371/journal.pone.0096289 (PMC4012980; doi:10.1371/journal.pone.0096289)
Supplement: Text S2 — Random effect regression results. (DOCX) [file pone.0096289.s006.docx]

**Text S2 Random effect regression results.**

Software: package plm in R (Swamy-Arora's transformation)

Significance Codes:

0 ‘***’ 0.001 ‘**’ 0.01 ‘*’ 0.05 ‘.’ 0.1 ‘ ’ 1

# Regression 1: White Males

Unbalanced Panel: Number of individuals=5514, Number of observations=8309

|  | **Estimate** | **St. Error** | **t-value** | **Pr(>\|t\|)** | **Sig. Code** |
| --- | --- | --- | --- | --- | --- |
| Intercept | 1.11E+01 | 2.08E+00 | 5.3568 | 8.70E-08 | *** |
| age^2 | -3.37E-03 | 7.57E-05 | -44.5402 | <2.20E-16 | *** |
| (age^2)*LowEd | 6.24E-04 | 1.81E-04 | 3.4495 | 0.000565 | *** |
| LowEd | -4.70E+00 | 9.81E-01 | -4.7881 | 1.71E-06 | *** |
| height | 5.31E-01 | 3.06E-02 | 17.3695 | <2.20E-16 | *** |
| weight | 3.64E-02 | 2.67E-03 | 13.6342 | <2.20E-16 | *** |
| wave2008 | -8.44E-01 | 2.06E-01 | -4.1007 | 4.16E-05 | *** |
| wave2010 | -9.48E-01 | 1.32E-01 | -7.1642 | 8.50E-13 | *** |
| wave2012 | -1.93E+00 | 2.08E-01 | -9.2754 | <2.20E-16 | *** |

Total Sum of Squares=208780; Residual Sum of Squares=112760

R-Squared= 0.46162; Adj. R-Squared=0.46112

F-statistic: 883.428 on 8 and 8300 DF, p-value: < 2.22e-16

Idiosyncratic and individual effects

|  | **Variance** | **Standard Deviation** | **Overall Variance Share** |
| --- | --- | --- | --- |
| Idiosyncratic effect | 13.178 | 3.630 | 0.269 |
| Individual effect | 35.837 | 5.986 | 0.731 |

# Regression 2: White Females

Unbalanced Panel: Number of individuals=6964, Number of observations=10446

|  | **Estimate** | **St. Error** | **t-value** | **Pr(>\|t\|)** | **Sig. Code** |
| --- | --- | --- | --- | --- | --- |
| Intercept | 5.75E+00 | 1.20E+00 | 4.7891 | 1.70E-06 | *** |
| age^2 | -2.02E-03 | 4.40E-05 | -45.7885 | < 2.2e-16 | *** |
| (age^2)*LowEd | -2.77E+00 | 5.41E-01 | -5.1204 | 3.10E-07 | *** |
| LowEd | 3.71E-04 | 1.01E-04 | 3.6817 | 0.000233 | *** |
| height | 3.76E-01 | 1.84E-02 | 20.4283 | < 2.2e-16 | *** |
| weight | 1.80E-02 | 1.46E-03 | 12.3191 | < 2.2e-16 | *** |
| wave2008 | -2.48E-01 | 1.19E-01 | -2.0777 | 0.037766 | * |
| wave2010 | -1.67E-01 | 7.98E-02 | -2.0896 | 0.036674 | * |
| wave2012 | -7.50E-01 | 1.21E-01 | -6.1953 | 6.04E-10 | *** |

Total Sum of Squares=110570; Residual Sum of Squares=65297

R-Squared= 0.41035; Adj. R-Squared=0.41

F-statistic: 904.545 on 8 and 10437 DF, p-value: < 2.22e-16

Idiosyncratic and individual effects

|  | **Variance** | **Standard Deviation** | **Overall Variance Share** |
| --- | --- | --- | --- |
| Idiosyncratic effect | 6.1158 | 2.473 | 0.294 |
| Individual effect | 14.70 | 14.70 | 0.706 |

# Regression 3: African-American Males

Unbalanced Panel: Number of individuals=1027, Number of observations=1368

|  | **Estimate** | **St. Error** | **t-value** | **Pr(>\|t\|)** | **Sig. Code** |
| --- | --- | --- | --- | --- | --- |
| Intercept | 1.64E+01 | 4.95E+00 | 3.3141 | 9.44E-04 | *** |
| age^2 | -2.84E-03 | 2.45E-04 | -11.5706 | < 2.2e-16 | *** |
| (age^2)*LowEd | 1.96E+00 | 1.91E+00 | 1.0257 | 3.05E-01 |  |
| LowEd | -3.24E-04 | 3.94E-04 | -0.8229 | 4.11E-01 |  |
| height | 3.36E-01 | 7.18E-02 | 4.6836 | 3.10E-06 | *** |
| weight | 5.43E-02 | 6.04E-03 | 8.9961 | < 2.2e-16 | *** |
| wave2008 | -3.12E-01 | 5.85E-01 | -0.5334 | 0.593818 |  |
| wave2010 | -4.99E-01 | 4.10E-01 | -1.2166 | 0.223982 |  |
| wave2012 | -1.48E+00 | 5.46E-01 | -2.7056 | 0.006902 | ** |

Total Sum of Squares=40471; Residual Sum of Squares=24245

R-Squared= 0.40348; Adj. R-Squared= 0.40082

F-statistic: 113.693 on 8 and 1359 DF, p-value: < 2.22e-16

Idiosyncratic and individual effects

|  | **Variance** | **Standard Deviation** | **Overall Variance Share** |
| --- | --- | --- | --- |
| Idiosyncratic effect | 17.594 | 4.194 | 0.311 |
| Individual effect | 39.017 | 6.246 | 0.689 |

**Regression 4: African-American Females**

Unbalanced Panel: Number of individuals=1643, Number of observations=2219

|  | **Estimate** | **St. Error** | **t-value** | **Pr(>\|t\|)** | **Sig. Code** |
| --- | --- | --- | --- | --- | --- |
| Intercept | 9.11E+00 | 2.86E+00 | 3.1907 | 1.44E-03 | ** |
| age^2 | -1.57E-03 | 1.28E-04 | -12.261 | < 2.2e-16 | *** |
| (age^2)*LowEd | -2.15E+00 | 1.06E+00 | -2.0421 | 4.13E-02 | * |
| LowEd | 3.39E-04 | 2.18E-04 | 1.5587 | 0.119221 |  |
| height | 3.05E-01 | 4.31E-02 | 7.0792 | 1.94E-12 | *** |
| weight | 1.73E-02 | 3.20E-03 | 5.4056 | 7.15E-08 | *** |
| wave2008 | -7.02E-02 | 3.20E-01 | -0.2191 | 0.8266 |  |
| wave2010 | -7.69E-01 | 2.29E-01 | -3.3628 | 0.000785 | *** |
| wave2012 | -1.06E+00 | 3.04E-01 | -3.4746 | 0.000522 | *** |

Total Sum of Squares=29955; Residual Sum of Squares=21038

R-Squared= 0.30218; Adj. R-Squared= 0.30095

F-statistic: 117.091 on 8 and 2210 DF, p-value: < 2.22e-16

Idiosyncratic and individual effects

|  | **Variance** | **Standard Deviation** | **Overall Variance Share** |
| --- | --- | --- | --- |
| Idiosyncratic effect | 9.281 | 3.046 | 0.336 |
| Individual effect | 18.379 | 4.287 | 0.664 |
