# Supplementary material for: Measuring the Speed of Aging across Population Subgroups
Source: PLoS One. 2014 May 7;9(5):e96289. doi: 10.1371/journal.pone.0096289 (PMC4012980; doi:10.1371/journal.pone.0096289)
Supplement: Text S3 — Tests for Selection. (DOCX) [file pone.0096289.s007.docx]

**Text S3 Tests for Selection.**

Heckman 2-step procedure

## Step 1: Probit selection equation

Sample: All people chosen *ex ante* for an enhanced face to face interview (hg_sample=3, see Text S1).

$y_{i}^{*}=Z_{i}\gamma+u_{i},$,

where hand-grip strength is observed (try = 1, see Text S1) for person *i* if $y_{i}^{*}>0$, $Z_{i}$ is a set of covariates for person *I,* $\gamma$ is a vector of parameters and $u_{i}$ a draw from a normal random variable with variance $\sigma^{2}$.

## Step 2: Outcome equation

We want to estimate the parameters $\beta$from the equation

$hg_{i}= X_{i}\beta+ \varepsilon_{i}$,

where $hg_{i}$ is the hand-grip strength of the *i-*th individual, $X_{i}$ is a set of covariates for hand-grip strength, and $\varepsilon$ and $\mu$have a bivariate normal distribution with correlation $\rho$.

In order to do this we add a variable to the hand-grip strength equation, the inverse of the Mills ratio, based on coefficients of the selection equation. Adjusted standard errors are computed and we test for the existence of selection effects by assessing the statistical significance of the coefficient on the inverse Mills ratio.

Since the bulk of the cases where hg_sample = 3 and try ≠ 1 arose because of people not wishing an interview at home, we used a dummy variable for whether or not the person was living alone as one of the *Z* variables. In the selection equation, we could use only variables that were available for everyone with hg_sample = 3. Since some people with hg_sample = 3 did not have face to face interviews, information on their weight and height was missing, and therefore, we could not include weight and height as variables in the selection equation.

We performed the selection tests separately for each wave, race, and gender combination. The result was 16 separate tests.

**1. Wave 2006, White Males**

2814 observations (591 censored and 2223 observed)

Probit selection equation:

|  | **Estimate** | **Std. Error** | **t value** | **Pr(>|t|)** | **Sig. Code** |
| --- | --- | --- | --- | --- | --- |
| Intercept | 1.637948 | 0.461675 | 3.548 | 0.000395 | *** |
| Living Alone | -1.01637 | 0.521532 | -1.949 | 0.051417 | . |
| Age | -0.01264 | 0.006368 | -1.985 | 0.047233 | * |
| LowEd | -0.43396 | 0.065276 | -6.648 | 3.56e-11 | *** |
| Living Alone*Low Ed | 0.017231 | 0.007234 | 2.382 | 0.017295 | * |

Outcome equation:

|  | **Estimate** | **Std. Error** | **t value** | **Pr(>|t|)** | **Sig. Code** |
| --- | --- | --- | --- | --- | --- |
| Intercept | -3.60779 | 4.320264 | -0.835 | 0.4037 |  |
| Age^2 | -0.00333 | 0.00015 | -22.12 | < 2e-16 | *** |
| LowEd | -4.1046 | 1.870895 | -2.194 | 0.0283 | * |
| Height | 0.795619 | 0.062186 | 12.794 | < 2e-16 | *** |
| Weight | 0.028074 | 0.00487 | 5.764 | 9.09e-09 | *** |
| (Age^2)*LowEd | 0.000797 | 0.000324 | 2.462 | 0.0139 | * |

Multiple R-Squared: 0.3521, Adjusted R-Squared: 0.3503

Error terms:

|  | **Estimate** | **Std. Error** | **t value** | **Pr(>|t|)** | **Sig. Code** |
| --- | --- | --- | --- | --- | --- |
| InvMillsRatio | -6.337 | 3.769 | -1.681 | 0.0928 | . |
| sigma | 8.207 |  |  |  |  |
| rho | -0.772 |  |  |  |  |

# **2. Wave 2006, White Females**

3525 observations (797 censored and 2728 observed)

Probit selection equation:

|  | **Estimate** | **Std. Error** | **t value** | **Pr(>|t|)** | **Sig. Code** |
| --- | --- | --- | --- | --- | --- |
| Intercept | 2.070397 | 0.288154 | 7.185 | 8.17e-13 | *** |
| Living Alone | -0.609033 | 0.388127 | -1.569 | 0.1167 |  |
| Age | -0.018524 | 0.003824 | -4.844 | 1.33e-06 | *** |
| LowEd | -0.306323 | 0.058291 | -5.255 | 1.57e-07 | *** |
| Living Alone*Low Ed | 0.01043 | 0.005378 | 1.939 | 0.0525 | . |

Outcome equation:

|  | **Estimate** | **Std. Error** | **t value** | **Pr(>|t|)** | **Sig. Code** |
| --- | --- | --- | --- | --- | --- |
| Intercept | -0.4170738 | 2.4700078 | -0.169 | 0.866 |  |
| Age^2 | -0.0019357 | 0.0001431 | -13.529 | < 2e-16 | *** |
| LowEd | -4.6511574 | 1.0513923 | -4.424 | 9.99e-06 | *** |
| Height | 0.4774832 | 0.0377766 | 12.64 | < 2e-16 | *** |
| Weight | 0.0142654 | 0.0026635 | 5.356 | 9.06e-08 | *** |
| (Age^2)*LowEd | 0.0007929 | 0.0001954 | 4.058 | 5.06e-05 | *** |

Multiple R-Squared: 0.3146, Adjusted R-Squared: 0.3131

Error terms:

|  | **Estimate** | **Std. Error** | **t value** | **Pr(>|t|)** | **Sig. Code** |
| --- | --- | --- | --- | --- | --- |
| InvMillsRatio | 0.30531 | 2.57833 | -0.118 | 0.906 |  |
| sigma | 4.63763 |  |  |  |  |
| rho | 0.06583 |  |  |  |  |

# **3. Wave 2006, African-American Males**

404 observations (132 censored and 272 observed)

Probit selection equation:

|  | **Estimate** | **Std. Error** | **t value** | **Pr(>|t|)** | **Sig. Code** |
| --- | --- | --- | --- | --- | --- |
| Intercept | 1.57201 | 0.9138 | 1.72 | 0.08617 | . |
| Living Alone | -1.66432 | 1.12359 | -1.481 | 1.39e-01 |  |
| Age | -0.01178 | 0.01299 | -0.907 | 0.36511 |  |
| LowEd | -0.38483 | 0.13718 | -2.805 | 5.28e-03 | ** |
| Living Alone*Low Ed | 0.0214 | 0.01601 | 1.337 | 1.82e-01 |  |

Outcome equation:

|  | **Estimate** | **Std. Error** | **t value** | **Pr(>|t|)** | **Sig. Code** |
| --- | --- | --- | --- | --- | --- |
| Intercept | -9.7348059 | 11.9771149 | -0.813 | 0.41684 |  |
| Age^2 | -0.0030318 | 0.0005358 | -5.658 | 2.96e-08 | *** |
| LowEd | 0.4882789 | 4.4226935 | 0.11 | 0.91215 |  |
| Height | 0.7948817 | 0.1692101 | 4.698 | 3.65e-06 | *** |
| Weight | 0.04275 | 0.0131424 | 3.253 | 0.00124 | ** |
| (Age^2)*LowEd | 0.0002346 | 0.0008073 | 0.291 | 0.77152 |  |

Multiple R-Squared: 0.3207, Adjusted R-Squared: 0.3053

Error terms:

|  | **Estimate** | **Std. Error** | **t value** | **Pr(>|t|)** | **Sig. Code** |
| --- | --- | --- | --- | --- | --- |
| InvMillsRatio | 4.9997 | 7.8709 | -0.635 | 0.526 |  |
| sigma | 8.1485 |  |  |  |  |
| rho | 0.6136 |  |  |  |  |

# **4. Wave 2006, African-American Females**

651 observations (195 censored and 456 observed)

Probit selection equation:

|  | **Estimate** | **Std. Error** | **t value** | **Pr(>|t|)** | **Sig. Code** |
| --- | --- | --- | --- | --- | --- |
| Intercept | 1.157919 | 0.493737 | 2.345 | 1.93E-02 | * |
| Living Alone | -0.954621 | 0.91076 | -1.048 | 0.295 |  |
| Age | -0.009303 | 0.007037 | -1.322 | 1.87E-01 |  |
| LowEd | -0.143521 | 0.109298 | -1.313 | 0.1896 |  |
| Living Alone*Low Ed | 0.017107 | 0.013506 | 1.267 | 0.2058 |  |

Outcome equation:

|  | **Estimate** | **Std. Error** | **t value** | **Pr(>|t|)** | **Sig. Code** |
| --- | --- | --- | --- | --- | --- |
| Intercept | 0.1565805 | 7.1116452 | 0.022 | 0.982441 |  |
| Age^2 | -0.0013055 | 0.0003592 | -3.635 | 0.000301 | *** |
| LowEd | -0.2089085 | 2.1923302 | -0.095 | 0.924114 |  |
| Height | 0.5478304 | 0.1022168 | 5.359 | 1.17E-07 | *** |
| Weight | -0.0049624 | 0.0070179 | -0.707 | 0.479758 |  |
| (Age^2)*LowEd | 0.0002224 | 0.0004254 | 0.523 | 0.601195 |  |

Multiple R-Squared: 0.1768, Adjusted R-Squared: 0.1658

Error terms:

|  | **Estimate** | **Std. Error** | **t value** | **Pr(>|t|)** | **Sig. Code** |
| --- | --- | --- | --- | --- | --- |
| InvMillsRatio | -8.502 | 5.983 | -1.421 | 0.156 |  |
| sigma | 7.996 |  |  |  |  |
| rho | -1.063 |  |  |  |  |

# **5. Wave 2008, White Males**

2526 observations (493 censored and 2033 observed)

Probit selection equation:

|  | **Estimate** | **Std. Error** | **t value** | **Pr(>|t|)** | **Sig. Code** |
| --- | --- | --- | --- | --- | --- |
| Intercept | 1.814323 | 0.483567 | 3.752 | 0.000179 | *** |
| Living Alone | -1.327851 | 0.551248 | -2.409 | 0.016076 | * |
| Age | -0.014934 | 0.006535 | -2.285 | 0.022394 | * |
| LowEd | -0.384306 | 0.070544 | -5.448 | 5.60e-08 | *** |
| Living Alone*Low Ed | 0.022286 | 0.007537 | 2.957 | 0.003137 | ** |

Outcome equation:

|  | **Estimate** | **Std. Error** | **t value** | **Pr(>|t|)** | **Sig. Code** |
| --- | --- | --- | --- | --- | --- |
| Intercept | 9.2772387 | 3.9765341 | 2.333 | 0.0197 | * |
| Age^2 | -0.0035908 | 0.0001485 | -24.184 | < 2e-16 |  |
| LowEd | -7.808795 | 1.9756693 | -3.952 | 7.95e-05 | *** |
| Height | 0.5935933 | 0.0573485 | 10.351 | < 2e-16 |  |
| Weight | 0.0306484 | 0.0050125 | 6.114 | 1.12e-09 | *** |
| (Age^2)*LowEd | 0.0014979 | 0.0003545 | 4.225 | 2.47e-05 | *** |

Multiple R-Squared: 0.3626, Adjusted R-Squared: 0.3607

Error terms:

|  | **Estimate** | **Std. Error** | **t value** | **Pr(>|t|)** | **Sig. Code** |
| --- | --- | --- | --- | --- | --- |
| InvMillsRatio | -4.1161 | 2.8992 | -1.42 | 0.156 |  |
| sigma | 7.6708 |  |  |  |  |
| rho | -0.5366 |  |  |  |  |

# **6. Wave 2008, White Females**

3284 observations (643 censored and 2641 observed)

Probit selection equation:

|  | **Estimate** | **Std. Error** | **t value** | **Pr(>|t|)** | **Sig. Code** |
| --- | --- | --- | --- | --- | --- |
| Intercept | 2.501162 | 0.296251 | 8.443 | < 2e-16 | *** |
| Living Alone | -1.471412 | 0.413959 | -3.554 | 0.000384 | *** |
| Age | -0.023925 | 0.003927 | -6.092 | 1.24e-09 | *** |
| LowEd | -0.097163 | 0.06195 | -1.568 | 0.116884 |  |
| Living Alone*Low Ed | 0.023838 | 0.00574 | 4.153 | 3.37e-05 | *** |

Outcome equation:

|  | **Estimate** | **Std. Error** | **t value** | **Pr(>|t|)** | **Sig. Code** |
| --- | --- | --- | --- | --- | --- |
| Intercept | 0.3993918 | 2.2761382 | 0.175 | 0.86072 |  |
| Age^2 | -0.002006 | 0.0001052 | -19.072 | < 2e-16 | *** |
| LowEd | -2.9577233 | 0.9934193 | -2.977 | 0.00293 | ** |
| Height | 0.4629134 | 0.0353914 | 13.08 | < 2e-16 | *** |
| Weight | 0.0148687 | 0.0027654 | 5.377 | 8.12e-08 | *** |
| (Age^2)*LowEd | 0.0003558 | 0.0001856 | 1.916 | 0.0554 | . |

Multiple R-Squared: 0.33, Adjusted R-Squared: 0.3285

Error terms:

|  | **Estimate** | **Std. Error** | **t value** | **Pr(>|t|)** | **Sig. Code** |
| --- | --- | --- | --- | --- | --- |
| InvMillsRatio | 0.24859 | 1.48873 | 0.167 | 0.867 |  |
| sigma | 4.76973 |  |  |  |  |
| rho | 0.05212 |  |  |  |  |

# **7. Wave 2008, African-American Males**

374 observations (98 censored and 276 observed)

Probit selection equation:

|  | **Estimate** | **Std. Error** | **t value** | **Pr(>|t|)** | **Sig. Code** |
| --- | --- | --- | --- | --- | --- |
| Intercept | 1.243565 | 0.854606 | 1.455 | 0.1465 |  |
| Living Alone | 0.504988 | 1.150351 | 0.439 | 0.6609 |  |
| Age | -0.007954 | 0.012086 | -0.658 | 0.5109 |  |
| LowEd | -0.351578 | 0.149491 | -2.352 | 0.0192 | * |
| Living Alone*Low Ed | -0.005062 | 0.016238 | -0.312 | 0.7554 |  |

Outcome equation:

|  | **Estimate** | **Std. Error** | **t value** | **Pr(>|t|)** | **Sig. Code** |
| --- | --- | --- | --- | --- | --- |
| Intercept | 9.0968168 | 13.3294263 | 0.682 | 0.49539 |  |
| Age^2 | -0.0024644 | 0.0014725 | -1.674 | 0.09508 | . |
| LowEd | 6.3700118 | 5.8022988 | 1.098 | 0.273 |  |
| Height | 0.5828262 | 0.1763119 | 3.306 | 0.00104 | ** |
| Weight | 0.0435844 | 0.014053 | 3.101 | 0.00208 | ** |
| (Age^2)*LowEd | 0.0000107 | 0.0007642 | 0.014 | 0.98883 |  |

Multiple R-Squared: 0.3206, Adjusted R-Squared: 0.3055

Error terms:

|  | **Estimate** | **Std. Error** | **t value** | **Pr(>|t|)** | **Sig. Code** |
| --- | --- | --- | --- | --- | --- |
| InvMillsRatio | -27.825 | 29.997 | -0.928 | 0.354 |  |
| sigma | 20.573 |  |  |  |  |
| rho | -1.353 |  |  |  |  |

# **8. Wave 2008, African-American Females**

618 observations (145 censored and 473 observed)

Probit selection equation:

|  | **Estimate** | **Std. Error** | **t value** | **Pr(>|t|)** | **Sig. Code** |
| --- | --- | --- | --- | --- | --- |
| Intercept | 1.195589 | 0.556137 | 2.15 | 0.032 | * |
| Living Alone | -0.75424 | 0.990234 | -0.762 | 0.447 |  |
| Age | -0.005065 | 0.007967 | -0.636 | 0.525 |  |
| LowEd | -0.16674 | 0.123302 | -1.352 | 0.177 |  |
| Living Alone*Low Ed | 0.008476 | 0.014509 | 0.584 | 0.559 |  |

Outcome equation:

|  | **Estimate** | **Std. Error** | **t value** | **Pr(>|t|)** | **Sig. Code** |
| --- | --- | --- | --- | --- | --- |
| Intercept | 4.9584815 | 6.7446351 | 0.735 | 0.462518 |  |
| Age^2 | -0.0019371 | 0.0003253 | -5.955 | 4.42e-09 | *** |
| LowEd | -7.4907164 | 2.5267242 | -2.965 | 0.00315 | ** |
| Height | 0.3343084 | 0.0890579 | 3.754 | 0.000191 | *** |
| Weight | 0.0224981 | 0.006836 | 3.291 | 0.001056 | ** |
| (Age^2)*LowEd | 0.0011619 | 0.0004707 | 2.469 | 0.013838 | * |

Multiple R-Squared: 0.2201, Adjusted R-Squared: 0.21

Error terms:

|  | **Estimate** | **Std. Error** | **t value** | **Pr(>|t|)** | **Sig. Code** |
| --- | --- | --- | --- | --- | --- |
| InvMillsRatio | 8.847 | 8.246 | 1.073 | 0.284 |  |
| sigma | 8.066 |  |  |  |  |
| rho | 1.097 |  |  |  |  |

# **9. Wave 2010, White Males**

2784 observations (683 censored and 2101 observed)

Probit selection equation:

|  | **Estimate** | **Std. Error** | **t value** | **Pr(>|t|)** | **Sig. Code** |
| --- | --- | --- | --- | --- | --- |
| Intercept | 2.021941 | 0.42807 | 4.723 | 2.43e-06 | *** |
| Living Alone | -1.380073 | 0.48439 | -2.849 | 0.00442 | ** |
| Age | -0.018864 | 0.005896 | -3.2 | 0.00139 | ** |
| LowEd | -0.346087 | 0.068735 | -5.035 | 5.08e-07 | *** |
| Living Alone*Low Ed | 0.020695 | 0.006711 | 3.084 | 0.00207 | ** |

Outcome equation:

|  | **Estimate** | **Std. Error** | **t value** | **Pr(>|t|)** | **Sig. Code** |
| --- | --- | --- | --- | --- | --- |
| Intercept | 2.485646 | 4.2707892 | 0.582 | 0.5606 |  |
| Age^2 | -0.0030689 | 0.000143 | -21.457 | < 2e-16 | *** |
| LowEd | -4.4561885 | 1.9764444 | -2.255 | 0.0242 | * |
| Height | 0.6719778 | 0.0599093 | 11.217 | < 2e-16 | *** |
| Weight | 0.024027 | 0.0049734 | 4.831 | 1.43e-06 | *** |
| (Age^2)*LowEd | 0.0005394 | 0.0003457 | 1.56 | 0.1188 |  |

Multiple R-Squared: 0.3358, Adjusted R-Squared: 0.3339

Error terms:

|  | **Estimate** | **Std. Error** | **t value** | **Pr(>|t|)** | **Sig. Code** |
| --- | --- | --- | --- | --- | --- |
| InvMillsRatio | -2.1868 | 4.0236 | -0.543 | 0.587 |  |
| sigma | 7.448 |  |  |  |  |
| rho | -0.2936 |  |  |  |  |

# **10. Wave 2010, White Females**

3488 observations (855 censored and 2633 observed)

Probit selection equation:

|  | **Estimate** | **Std. Error** | **t value** | **Pr(>|t|)** | **Sig. Code** |
| --- | --- | --- | --- | --- | --- |
| Intercept | 2.350682 | 0.280152 | 8.391 | < 2e-16 | *** |
| Living Alone | -1.459432 | 0.374392 | -3.898 | 9.88e-05 | *** |
| Age | -0.023031 | 0.003718 | -6.195 | 6.49e-10 | *** |
| LowEd | -0.262754 | 0.061027 | -4.306 | 1.71e-05 | *** |
| Living Alone*Low Ed | 0.02187 | 0.005188 | 4.216 | 2.55e-05 | *** |

Outcome equation:

|  | **Estimate** | **Std. Error** | **t value** | **Pr(>|t|)** | **Sig. Code** |
| --- | --- | --- | --- | --- | --- |
| Intercept | 2.3465011 | 2.378697 | 0.986 | 0.324 |  |
| Age^2 | -0.0019818 | 0.0001108 | -17.89 | < 2e-16 | *** |
| LowEd | -2.7222185 | 1.0903477 | -2.497 | 0.0126 | * |
| Height | 0.4239195 | 0.0361731 | 11.719 | < 2e-16 | *** |
| Weight | 0.0141941 | 0.0027085 | 5.241 | 1.70E-07 | *** |
| (Age^2)*LowEd | 0.0003955 | 0.0002066 | 1.914 | 0.0557 | . |

Multiple R-Squared: 0.3042, Adjusted R-Squared: 0.3026

Error terms:

|  | **Estimate** | **Std. Error** | **t value** | **Pr(>|t|)** | **Sig. Code** |
| --- | --- | --- | --- | --- | --- |
| InvMillsRatio | 1.5595 | 1.9145 | 0.815 | 0.415 |  |
| sigma | 4.894 |  |  |  |  |
| rho | 0.3187 |  |  |  |  |

# **11. Wave 2010, African-American Males**

545 observations (150 censored and 395 observed)

Probit selection equation:

|  | **Estimate** | **Std. Error** | **t value** | **Pr(>|t|)** | **Sig. Code** |
| --- | --- | --- | --- | --- | --- |
| Intercept | 2.166058 | 0.71566 | 3.027 | 0.00259 | ** |
| Living Alone | -0.252819 | 0.904069 | -0.28 | 0.77986 |  |
| Age | -0.02457 | 0.010613 | -2.315 | 0.02099 | * |
| LowEd | -0.039988 | 0.126069 | -0.317 | 0.75122 |  |
| Living Alone*Low Ed | 0.005702 | 0.013364 | 0.427 | 0.6698 |  |

Outcome equation:

|  | **Estimate** | **Std. Error** | **t value** | **Pr(>|t|)** | **Sig. Code** |
| --- | --- | --- | --- | --- | --- |
| Intercept | 30.9841217 | 9.2658372 | 3.344 | 0.000884 | *** |
| Age^2 | -0.0012865 | 0.0014403 | -0.893 | 0.372158 |  |
| LowEd | 2.228541 | 3.3677721 | 0.662 | 0.508434 |  |
| Height | 0.1699201 | 0.1311941 | 1.295 | 0.195819 |  |
| Weight | 0.0386473 | 0.010771 | 3.588 | 0.000364 | *** |
| (Age^2)*LowEd | -0.0004708 | 0.0006713 | -0.701 | 0.483398 |  |

Multiple R-Squared: 0.1891, Adjusted R-Squared: 0.1766

Error terms:

|  | **Estimate** | **Std. Error** | **t value** | **Pr(>|t|)** | **Sig. Code** |
| --- | --- | --- | --- | --- | --- |
| InvMillsRatio | -16.176 | 17.607 | -0.919 | 0.359 |  |
| sigma | 13.491 |  |  |  |  |
| rho | -1.199 |  |  |  |  |

**12. Wave 2010, African-American Females**

888 observations (235 censored and 653 observed)

Probit selection equation:

|  | **Estimate** | **Std. Error** | **t value** | **Pr(>|t|)** | **Sig. Code** |
| --- | --- | --- | --- | --- | --- |
| Intercept | 2.58943 | 0.396373 | 6.533 | 1.09e-10 | *** |
| Living Alone | -1.102769 | 0.772137 | -1.428 | 0.1536 |  |
| Age | -0.027806 | 0.005735 | -4.849 | 1.47e-06 | *** |
| LowEd | -0.209687 | 0.100667 | -2.083 | 0.0375 | * |
| Living Alone*Low Ed | 0.016005 | 0.011629 | 1.376 | 0.1691 |  |

Outcome equation:

|  | **Estimate** | **Std. Error** | **t value** | **Pr(>|t|)** | **Sig. Code** |
| --- | --- | --- | --- | --- | --- |
| Intercept | 7.08e-01 | 5.603e+00 | 0.126 | 0.89954 |  |
| Age^2 | -1.841e-03 | 6.967e-04 | -2.642 | 0.00838 | ** |
| LowEd | -1.232e+00 | 1.855e+00 | -0.664 | 0.50672 |  |
| Height | 4.178e-01 | 8.409e-02 | 4.968 | 8.12e-07 | *** |
| Weight | 1.293e-02 | 5.524e-03 | 2.341 | 0.01948 | * |
| (Age^2)*LowEd | 2.512e-05 | 3.959e-04 | 0.063 | 0.94942 |  |

Multiple R-Squared: 0.1609, Adjusted R-Squared: 0.1531

Error terms:

|  | **Estimate** | **Std. Error** | **t value** | **Pr(>|t|)** | **Sig. Code** |
| --- | --- | --- | --- | --- | --- |
| InvMillsRatio | 6.0192 | 8.2492 | 0.73 | 0.466 |  |
| sigma | 6.898 |  |  |  |  |
| rho | 0.8726 |  |  |  |  |

**13. Wave 2012, White Males**

2506 observations (469 censored and 2037 observed)

Probit selection equation:

|  | **Estimate** | **Std. Error** | **t value** | **Pr(>|t|)** | **Sig. Code** |
| --- | --- | --- | --- | --- | --- |
| Intercept | 1.862584 | 0.438602 | 4.247 | 2.25e-05 | *** |
| Living Alone | -0.600138 | 0.508191 | -1.181 | 0.2377 |  |
| Age | -0.014181 | 0.006006 | -2.361 | 0.0183 | * |
| LowEd | -0.372336 | 0.074514 | -4.997 | 6.23e-07 | *** |
| Living Alone*Low Ed | 0.010297 | 0.007 | 1.471 | 0.1415 |  |

Outcome equation:

|  | **Estimate** | **Std. Error** | **t value** | **Pr(>|t|)** | **Sig. Code** |
| --- | --- | --- | --- | --- | --- |
| Intercept | 6.6864362 | 4.2686351 | 1.566 | 0.1174 |  |
| Age^2 | -0.003014 | 0.0002037 | -14.793 | < 2e-16 | *** |
| LowEd | -3.539904 | 1.9625654 | -1.804 | 0.0714 | . |
| Height | 0.612794 | 0.0591907 | 10.353 | < 2e-16 | *** |
| Weight | 0.0279216 | 0.0051578 | 5.413 | 6.77e-08 | *** |
| (Age^2)*LowEd | 0.000641 | 0.0003252 | 1.971 | 0.0489 | * |

Multiple R-Squared: 0.3544, Adjusted R-Squared: 0.3525

Error terms:

|  | **Estimate** | **Std. Error** | **t value** | **Pr(>|t|)** | **Sig. Code** |
| --- | --- | --- | --- | --- | --- |
| InvMillsRatio | -9.852 | 6.71 | -1.468 | 0.142 |  |
| sigma | 9.663 |  |  |  |  |
| rho | -1.02 |  |  |  |  |

**14. Wave 2012, White Females**

3216 observations (665 censored and 2551 observed)

Probit selection equation:

|  | **Estimate** | **Std. Error** | **t value** | **Pr(>|t|)** | **Sig. Code** |
| --- | --- | --- | --- | --- | --- |
| Intercept | 2.217838 | 0.285186 | 7.777 | 9.95e-15 | *** |
| Living Alone | -0.865351 | 0.397096 | -2.179 | 0.02939 | * |
| Age | -0.020137 | 0.003814 | -5.28 | 1.38e-07 | *** |
| LowEd | -0.242859 | 0.063166 | -3.845 | 0.000123 | *** |
| Living Alone*Low Ed | 0.014817 | 0.005517 | 2.686 | 0.007277 | ** |

Outcome equation:

|  | **Estimate** | **Std. Error** | **t value** | **Pr(>|t|)** | **Sig. Code** |
| --- | --- | --- | --- | --- | --- |
| Intercept | 3.1786541 | 2.2091787 | 1.439 | 0.15 |  |
| Age^2 | -0.001856 | 0.000118 | -15.724 | < 2e-16 | *** |
| LowEd | -1.2987655 | 1.0515846 | -1.235 | 0.217 |  |
| Height | 0.4012182 | 0.0338331 | 11.859 | < 2e-16 | *** |
| Weight | 0.0183534 | 0.0026885 | 6.827 | 1.04e-11 | *** |
| (Age^2)*LowEd | 0.0001316 | 0.0001953 | 0.674 | 0.501 |  |

Multiple R-Squared: 0.3332, Adjusted R-Squared: 0.3316

Error terms:

|  | **Estimate** | **Std. Error** | **t value** | **Pr(>|t|)** | **Sig. Code** |
| --- | --- | --- | --- | --- | --- |
| InvMillsRatio | -1.8361 | 1.9745 | -0.93 | 0.352 |  |
| sigma | 4.8717 |  |  |  |  |
| rho | -0.3769 |  |  |  |  |

**15. Wave 2012, African-American Males**

546 observations (106 censored and 440 observed)

Probit selection equation:

|  | **Estimate** | **Std. Error** | **t value** | **Pr(>|t|)** | **Sig. Code** |
| --- | --- | --- | --- | --- | --- |
| Intercept | 2.607094 | 0.75433 | 3.456 | 0.000592 | *** |
| Living Alone | 0.102052 | 0.947032 | 0.108 | 0.914227 |  |
| Age | -0.025357 | 0.011234 | -2.257 | 0.024401 | * |
| LowEd | -0.07984 | 0.147036 | -0.543 | 0.587359 |  |
| Living Alone*Low Ed | -0.002235 | 0.013951 | -0.16 | 0.872795 |  |

Outcome equation:

|  | **Estimate** | **Std. Error** | **t value** | **Pr(>|t|)** | **Sig. Code** |
| --- | --- | --- | --- | --- | --- |
| Intercept | 15.2681956 | 11.1972745 | 1.364 | 0.1733 |  |
| Age^2 | -0.0062177 | 0.0084424 | -0.736 | 0.4618 |  |
| LowEd | 1.7726557 | 2.2693492 | 0.781 | 0.4351 |  |
| Height | 0.3440078 | 0.1477426 | 2.328 | 0.0203 | * |
| Weight | 0.0496034 | 0.011954 | 4.15 | 3.88e-05 | *** |
| (Age^2)*LowEd | -0.0006928 |  |  |  |  |

Multiple R-Squared: 0.2418, Adjusted R-Squared: 0.2313

Error terms:

|  | **Estimate** | **Std. Error** | **t value** | **Pr(>|t|)** | **Sig. Code** |
| --- | --- | --- | --- | --- | --- |
| InvMillsRatio | 45.751 | 102.982 | 0.444 | 0.657 |  |
| sigma | 29.83 |  |  |  |  |
| rho | 1.534 |  |  |  |  |

**16. Wave 2012, African-American Females**

859 observations (198 censored and 661 observed)

Probit selection equation:

|  | **Estimate** | **Std. Error** | **t value** | **Pr(>|t|)** | **Sig. Code** |
| --- | --- | --- | --- | --- | --- |
| Intercept | 2.109738 | 0.418869 | 5.037 | 5.79e-07 | *** |
| Living Alone | -0.308781 | 0.823111 | -0.375 | 0.707651 |  |
| Age | -0.021145 | 0.006174 | -3.425 | 0.000645 | *** |
| LowEd | 0.022432 | 0.111947 | 0.2 | 0.841229 |  |
| Living Alone*Low Ed | 0.006354 | 0.012411 | 0.512 | 0.608839 |  |

Outcome equation:

|  | **Estimate** | **Std. Error** | **t value** | **Pr(>|t|)** | **Sig. Code** |
| --- | --- | --- | --- | --- | --- |
| Intercept | 5.7958151 | 4.8886983 | 1.186 | 0.236131 |  |
| Age^2 | -0.0012438 | 0.0006081 | -2.045 | 0.041121 | * |
| LowEd | -2.3100425 | 1.8746201 | -1.232 | 0.21819 |  |
| Height | 0.304138 | 0.0743743 | 4.089 | 4.74e-05 | *** |
| Weight | 0.0199845 | 0.0055859 | 3.578 | 0.000366 | *** |
| (Age^2)*LowEd | 0.0003389 | 0.0003951 | 0.858 | 0.391361 |  |

Multiple R-Squared: 0.137, Adjusted R-Squared: 0.1291

Error terms:

|  | **Estimate** | **Std. Error** | **t value** | **Pr(>|t|)** | **Sig. Code** |
| --- | --- | --- | --- | --- | --- |
| InvMillsRatio | 1.4714 | 8.4159 | 0.175 | 0.861 |  |
| sigma | 5.4424 |  |  |  |  |
| rho | 0.2704 |  |  |  |  |
